# Murine Lung Cancer Increases CD4+ T Cell Apoptosis and Decreases Gut Proliferative Capacity in Sepsis

**DOI:** 10.1371/journal.pone.0149069

**Published:** 2016-03-28

**Authors:** John D. Lyons, Rohit Mittal, Katherine T. Fay, Ching-Wen Chen, Zhe Liang, Lindsay M. Margoles, Eileen M. Burd, Alton B. Farris, Mandy L. Ford, Craig M. Coopersmith

**Affiliations:** 1 Department of Surgery and Emory Critical Care Center, Emory University School of Medicine, Atlanta, GA, United States of America; 2 Department of Internal Medicine and Emory Critical Care Center, Emory University School of Medicine, Atlanta, GA, United States of America; 3 Department of Pathology and Laboratory Medicine, Emory University School of Medicine, Atlanta, GA, United States of America; 4 Department of Surgery and Emory Transplant Center, Emory University School of Medicine, Atlanta, GA, United States of America; University of Florida, UNITED STATES

## Abstract

**Background:**

Mortality is significantly higher in septic patients with cancer than in septic patients without a history of cancer. We have previously described a model of pancreatic cancer followed by sepsis from *Pseudomonas aeruginosa* pneumonia in which cancer septic mice have higher mortality than previously healthy septic mice, associated with increased gut epithelial apoptosis and decreased T cell apoptosis. The purpose of this study was to determine whether this represents a common host response by creating a new model in which both the type of cancer and the model of sepsis are altered.

**Methods:**

C57Bl/6 mice received an injection of 250,000 cells of the lung cancer line LLC-1 into their right thigh and were followed three weeks for development of palpable tumors. Mice with cancer and mice without cancer were then subjected to cecal ligation and puncture and sacrificed 24 hours after the onset of sepsis or followed 7 days for survival.

**Results:**

Cancer septic mice had a higher mortality than previously healthy septic mice (60% vs. 18%, p = 0.003). Cancer septic mice had decreased number and frequency of splenic CD4+ lymphocytes secondary to increased apoptosis without changes in splenic CD8+ numbers. Intestinal proliferation was also decreased in cancer septic mice. Cancer septic mice had a higher bacterial burden in the peritoneal cavity, but this was not associated with alterations in local cytokine, neutrophil or dendritic cell responses. Cancer septic mice had biochemical evidence of worsened renal function, but there was no histologic evidence of renal injury.

**Conclusions:**

Animals with cancer have a significantly higher mortality than previously healthy animals following sepsis. The potential mechanisms associated with this elevated mortality differ significantly based upon the model of cancer and sepsis utilized. While lymphocyte apoptosis and intestinal integrity are both altered by the combination of cancer and sepsis, the patterns of these alterations vary greatly depending on the models used.

## Introduction

Sepsis is the leading causes of death among critically ill patients in the United States with between 230,000 and 370,000 people dying of the disease annually [1]. Patients with malignancy are nearly ten times more likely to develop sepsis than the general population [2], and cancer represents the most common co-morbidity in septic patients[3–5]. Sepsis is also the leading cause of ICU admission in patients with cancer[6,7]. Importantly, cancer is also the co-morbidity associated with the highest risk of death in sepsis, with hospital mortality exceeding 50% in patients with cancer and either severe sepsis or septic shock[5,7–9].

The etiology behind the increased mortality seen in cancer patients who develop sepsis compared to previously healthy patients who develop sepsis is multifactorial[2,10]. While some deaths are related to immunosuppression caused by cancer treatment such as chemotherapy or radiation, others are likely related to a reduced ability of the host to appropriately respond to infection in the setting of chronic systemic changes related to underlying malignancy. Animal models of cancer, in isolation, demonstrate that not only is the tumor microenvironment altered, but that systemic T cell exhaustion and generalized immune suppression are also induced by cancer[11]. Further, the host response to a non-lethal infection is markedly altered following cancer, with phenotypic exhaustion in T cells associated with increasing expression of co-inhibitory receptors[12].

There are numerous similarities in the host response to both cancer and sepsis[13]. In an attempt to understand why hosts with cancer have increased mortality following sepsis compared to previously healthy hosts, we have described a model of pancreatic cancer followed by sepsis from *Pseudomonas aeruginosa* pneumonia[14]. Mortality was higher in cancer septic mice than previously healthy mice and this was associated with a decrease in T lymphocyte apoptosis and an increase in both gut epithelial apoptosis and bacteremia. Interestingly, preventing lymphocyte apoptosis—a strategy associated with uniform success in other pre-clinical models of sepsis—was associated with increased mortality in cancer septic mice[15].

Despite having a greater understanding of the pathophysiology of sepsis than ever before[16–18], there has been a remarkable inability to translate preclinical models of sepsis into effective treatments at the bedside, where management is generally supportive yet non-selective, with the exception of targeted antimicrobial therapy[19]. One reason (of many) for the failure of pre-clinical trials to translate into effective therapies for sepsis is that animal studies are performed in a homogenous previously healthy population, whereas human studies are performed on heterogeneous patients frequently with multiple co-morbidities. As such, we sought to determine whether our previous pre-clinical findings in cancer and sepsis would be generalizable if we altered both the type of cancer and the model of sepsis. To examine this, we developed a new clinically relevant model of lung cancer followed by sepsis induced by cecal ligation and puncture.

## Materials and Methods

### Animals

Male and female C57BL/6 mice were used in all experiments, with gender matching between experimental and control groups. Animals were 6–8 weeks of age prior to initiation of experiments. A subset of animals were then injected with tumor cells (details below) and all mice were then watched for an additional three weeks before a subset were subjected to cecal ligation and puncture (CLP, also detailed below) at which time they were watched for 1–7 days depending on whether they were used for non-survival or survival experiments. Thus animals were a minimum of 9 weeks old and a maximum of 12 weeks old at time of sacrifice. Experiments were performed in accordance with the National Institutes of Health Guidelines for the Use of Laboratory Animals and were approved by the Institutional Animal Care and Use Committee at Emory University School of Medicine (Protocol DAR-2001875-082815BN). All animals were housed in an approved university animal facility and were given free access to food and water throughout. Animals that were injected with tumor cells were monitored to ensure that tumors did not ulcerate and did not impede animal ambulation according to the Emory IACUC guidelines for tumor burden. Following CLP, all animals received buprenorphine post-operatively in an attempt to minimize animal suffering. For non-survival studies, animals were sacrificed 24 hours post-operatively via asphyxiation by CO2 or exsanguination under deep ketamine anesthesia. A different subset of animals was followed for survival for 7 days post-operatively. During this survival experiment, animals were checked twice daily. In addition to observing the same endpoints outlined above surrounding tumor growth, animals were also checked to determine if they were moribund related to operation. Animals that either met tumor endpoints or were moribund were sacrificed using humane endpoints. Moribund animals were identified as follows: a) surgical complications unresponsive to immediate intervention (wound dehiscence, bleeding, infection), b) medical conditions unresponsive to treatment such as self-mutilation, severe respiratory distress, icterus, major organ failure or intractable diarrhea, or c) clinical or behavioral signs unresponsive to appropriate intervention persisting for 1 day including significant inactivity, labored breathing, sunken eyes, hunched posture, piloerection/matted fur, one or more unresolving skin ulcers, and abnormal vocalization when handled. Animals that survived 7 days post-operatively were sacrificed at the conclusion of this experiment using asphyxiation by CO2.

### Cancer model

A murine lung carcinoma cell line (LLC1, American Type Culture Collection, Manassas, VA) was cultured in RPMI 1640 medium supplemented with 10% fetal bovine serum, 1% glutamine, 1% penicillin-streptomycin and 1%4-(2-hydroxyethyl)-1-piperazineethanesulfonic acid. The rationale for using a lung cancer cell line is that lung cancer has the second highest mortality for solid tumors in septic patients (2) (pancreatic cancer is the highest and was used for our prior experiments in sepsis and cancer). After expansion, cancer cells were dissociated from growth flasks via incubation with 0.25% trypsin, washed, centrifuged for 10 minutes at 1500 RPM and then re-suspended in phosphate buffered solution (PBS) to a final concentration of 250,000 cells per 0.2 ml (live cells selected via trypan blue examination). Mice randomized to receive cancer had a single subcutaneous injection of 250,000 tumor cells along the right inner thigh (cancer group) and were followed for 3 weeks prior to CLP to allow for tumor growth. Control mice were unmanipulated and thus had no intervention prior to CLP (previously healthy group) but were watched for an identical time as cancer mice so animals would be age matched.

### Sepsis model and experimental groups

A subset of cancer mice and previously healthy mice were then subjected to CLP, an established model of polymicrobial peritonitis[20]. Briefly, under isoflurane anesthesia, a small midline abdominal incision was made, and the cecum was exteriorized and ligated below the ileocecal valve, avoiding intestinal obstruction. The cecum was punctured twice with a 25 gauge needle and squeezed gently to extrude a small amount of stool. After placing the cecum back in the abdomen, the abdominal wall was closed in layers. Immediately following CLP, mice received subcutaneous injections of a) fluids (1ml of 0.9% saline) to account for insensible losses, b) antibiotics (50 mg/kg of ceftriaxone, Sigma-Aldrich, St. Louis, MO and 35 mg/kg metronidazole, Apotex Corp, Weston, FL) to mimic the clinical scenario where septic patients receive antimicrobial therapy and c) pain medication (0.1 mg/kg buprenex, McKesson Medical, San Francisco, CA) to minimize pain and suffering. Animals were either followed 7 days for survival or sacrificed at 24 hours for sample collection. Antibiotics were re-dosed at 12, 24 and 36 hours after surgery in survival studies.

We have previously published an extensive immunological assessment of lung cancer in unmanipulated mice [11] so did not repeat those studies. However, we did not previously have data on many of the outcomes assayed in this study and so performed experiments in both septic and non-septic animals where appropriate, in order to understand the impact of cancer in isolation, sepsis in isolation, and the combination of cancer and sepsis. The following is the terminology used for each experimental group: a) unmanipulated (mice that received neither cancer nor sepsis), b) cancer (mice that received tumor cell injection alone), c) previously healthy septic (mice that underwent CLP alone without prior intervention), and d) cancer septic (mice with tumor cell injection followed three weeks later by CLP).

### Leukocyte analysis

Phenotypic flow cytometric analysis of leukocytes was performed on processed cellular suspensions of splenocytes (whole spleens removed at time of sacrifice) or peritoneal fluid (2.5ml PBS injected into peritoneum and withdrawn after 5 seconds of gentle agitation). The number of cells per ml of suspension was calculated utilizing a Nexcelom Auto Cellometer, the results of which were used to determine absolute cell numbers. The following antibodies were used to stain cells prior to analysis: for peritoneal fluid, anti-GR1.1 FITC (BD Bioscience, San Jose, CA), anti-Cd11b PerCP (BioLegend, San Diego, CA), anti-Cd11c PeCy7 (eBioscience, San Diego, CA), and anti-MHC II APC Cy7 (eBioscience); for splenocyte phenotyping, anti-F4/80 PerCP (BioLegend), anti-Cd11c PeCy7 (eBioscience), anti-Cd11b APC (eBioscience), anti-B220 Alexa700 (BD Bioscience), and anti-MHC II APC Cy7 (eBioscience).

To determine the frequency of apoptotic lymphocytes, splenocytes were collected from sacrificed animals and processed to a suspension of 1x10^7^cells/ml, and 1x10^6^ splenocytes were then processed using a commercially available Annexin V and 7-AAD kit (BioLegend) following manufacturer’s instructions. Cells were then stained with anti-CD4-PO (Invitrogen, Carlsbad, CA), anti-CD8-PB (eBioscience) to determine the frequency of pro-apoptotic CD4 and CD8 T cells. A gating strategy excluded dead cells staining positive for 7-AAD from analysis.

For samples undergoing intracellular cytokine staining, 1x 10^6^ splenocytes were plated into a 96-well plate. Cells were suspended and incubated in RPMI 1640 culture medium and stimulated for four hours utilizing phorbol 12-myristate 13-acetate (30ng/mL) and ionomycin (400ng/mL) with 10μg/mL of Brefeldin A at 37°C. After stimulation, the cells were stained with anti-CD4 PacBlue (BD Bioscience), anti-CD8 PacOrange (Life Technologies Carlsbad, CA), anti-IL-2 FITC (BD Bioscience), anti-IL-4 PE (eBioscience), anti-CD44 PerCP (BioLegend), anti-CCR4 PeCy7 (BioLegend), anti-CXCR3 APC (eBioscience), and anti-IFNy Alexa700 (BD Bioscience). An LSR II flow cytometer (BD Biosciences) was used to run all samples and FlowJo 10.0.8r1 software (Tree Star, San Carlos, CA) was used to analyze all data.

### Intestinal permeability

At 19 hours following CLP, mice were gavaged with 0.5 ml of fluorescein isothiocyanate-dextran (FD4) at a concentration of 22mg/ml in PBS (Sigma-Aldrich)[21,22]. Plasma collected at time of sacrifice 5 hour later was then diluted with an equal volume of PBS, and FD4 concentration was determined by flourospectrometry with excitation/emission wavelengths of 485/520 nm with a standard curve of serial dilutions (BioTex Synergy HT, Winooski, VT).

### Intestinal proliferation

Proliferating intestinal epithelial cells were stained with 5-Bromo-2’deoxyuridine (BrdU). At 90 minutes prior to sacrifice, mice received intraperitoneal injections of BrdU (5mg/ml in 0.9% saline, Sigma-Aldrich) to label S-phase cells [23,24]. Jejunal tissue was then fixed in 10% formalin for 24 hours before being embedded in paraffin and slide-mounted in 5μm sections. Slides were then deparaffinized, rehydrated, and incubated in 1% hydrogen peroxide for 15 minutes before being heated in a pressure cooker in antigen decloaker (Biocare Medical, Concord, CA) for 45 minutes. Protein block (Dako, Carpinteria, CA) was performed for 30 minutes at room temperature and slides were incubated overnight at 4°C with rat monoclonal Anti-BrdU (1:500; Accurate Chemical and Scientific, Westbury, NJ). Samples were then incubated with goat anti-rat antibody (1:500; Accurate Chemical & Scientific) and streptavidin horseradish peroxidase (1:500; Dako), each for an hour at room temperature. Diaminobenzidine (DAB) was used to develop slides for 2–3 minutes, and counterstaining was performed with hematoxylin. BrdU-positive cells were quantified in 100 contiguous, well-oriented intestinal crypts.

### Intestinal apoptosis

Apoptosis of intestinal epithelial cells was quantified using two complementary techniques: active caspase-3 staining and morphologic analysis of hematoxylin-eosin stained sections[25,26]. Sections were treated as above with antigen decloaker and were then blocked with 20% normal goat serum (Vector Laboratories, Burlingame, CA). Slides were incubated overnight at 4°C with rabbit anti-caspase-3 (1:100; Cell Signaling, Beverly, MA), and then with goat anti-rabbit biotinylated antibody (1:500; Vector Laboratories) and streptavidin horseradish peroxidase (1:500; Dako) for one hour each at room temperature. Slides were developed with DAB and then counterstained. Caspase-3 positive cells were counted in 100 contiguous intestinal crypts.

Apoptotic cells were identified on hematoxylin-eosin-stained sections by identifying characteristic morphological changes including cell shrinkage with condensed and fragmented nuclei and quantifying them in 100 contiguous crypts.

### Villus length

Villus length was measured as the distance in μm from the crypt neck to the villus tip in 12 consecutive well-oriented jejunal villi using Nikon Elements imaging software- EIS-Elements BR 3.10 (Nikon Instruments, Melville, NY).

### Bacterial cultures

Quantitative cultures of whole blood and peritoneal fluid were prepared from serial 10-fold dilutions of samples in sterile 0.9% saline. A 100 μl aliquot of undiluted sample and each dilution from 10^−1^ to 10^−3^ was plated on blood agar plates (Remel, Lenexa, KS) and incubated at 35°C in a 5% CO_2_ atmosphere for 24 hours. Colony counts were obtained from plates containing fewer than 300 colonies. The number of colony-forming units (CFUs) per ml of original sample was determined by multiplying the number of colonies by the reciprocal of the dilution counted and adjusted for the volume of sample plated.

### Cytokines

Whole blood, peritoneal fluid and bronchoalveolar lavage (BAL) fluid were collected and then centrifuged at 10,000 RPM for 10 minutes. The supernatant from each was then collected and analyzed for cytokine concentrations using a 6-plex cytokine bead array according to manufacturer instructions (Bio-Rad Laboratories, Hercules, CA).

### Renal function

Whole blood was centrifuged at 10,000 RPM for 10 minutes. Serum creatinine was then measured using a creatinine microplate assay (Oxford Biomedical Research, Rochester Hills, MI) while blood urea nitrogen (BUN) was determined using a urea nitrogen colorimetric detection kit (Arbor Assays, Ann Arbor, MI). Whole kidneys were removed and fixed in 10% formalin for 24 hours before paraffin fixation and sectioning. After hematoxylin-eosin staining, sections were assessed for injury by a pathologist blinded to sample identity (ABF). Animal weights were measured at time of CLP and immediately prior to sacrifice to assess potential weight loss due to dehydration.

### Liver injury

Liver injury was evaluated by both serum liver enzymes and histology. Alanine aminotransferase (ALT) was measured on a Beckman AU480 chemistry auto-analyzer (Beckman Diagnostics, LaBrea, CA) following manufacturer instructions. In addition, portions of whole liver were removed and fixed in 10% formalin for 24 hours prior to paraffin embedding. Sections were then slide-mounted and stained with hematoxylin-eosin for analysis by a pathologist (ABF) blinded to sample identity.

### Lung injury

For histologic analysis, lungs of animals were flushed with 1ml of 10% formalin, and sections were then removed and fixed in 10% formalin for 24 hours prior to being embedded in paraffin. Lung sections were then stained with hematoxylin-eosin and examined by a pathologist (ABF) blinded to sample identity.

Myeloperoxidase (MPO) activity was also assessed in BAL fluid. The trachea was irrigated with 1 ml of PBS, and fluid was then withdrawn and centrifuged at 10,000 RPM for 10 minutes. Substrate buffer containing 0.0005% hydrogen peroxide and O-dianisidine was added to the supernatant, and MPO activity was assayed over 6 minutes at wavelength 460 (BioTek Synergy HT, Winooski, VT). MPO activity was calculated as optical density/minute per μl of BAL fluid.

Lung fluid protein concentration was measured by analyzing samples treated with protein assay reagent (Thermo Scientific, Rockford, IL) at 660 nm in conjunction with a standard curve of bovine serum albumin.

### Complete blood counts

Whole blood collected at time of sacrifice was collected in anti-coagulant-lined blood tubes and was analyzed for hemoglobin concentration, leukocyte count, and platelet count on a Heska HemaTrue^®^ veterinary hematology analyzer per manufacturer guidelines (Heska, Loveland, CO).

### Statistics

All data were analyzed using the statistical software program Prism 6.0 (GraphPad, San Diego, CA) and are presented as mean ± SEM. Data were tested for Gaussian distribution using the D'Agostino-Pearson omnibus normality test. Two -way comparisons on data with a Gaussian distribution were performed using the Student’s t-test. Two-way comparisons on data that did not have a Gaussian distribution were performed using the Mann-Whitney test. Multi-group comparisons were analyzed via one-way ANOVA, followed by the Tukey post-test. Survival was analyzed using the Log-Rank test. A p value of <0.05 was considered to be statistically significant throughout.

## Results

Mice that received subcutaneous injections of murine lung cancer cells developed well-circumscribed solitary tumors at the site of injection three weeks later (average size 1.5 cm in diameter). Post-mortem review of lung histology demonstrated microscopic metastatic disease in some animals, although no gross tumor spread was seen in any animals at time of sacrifice.

### The presence of pre-existing lung cancer worsens mortality following sepsis

**Fig 1:**
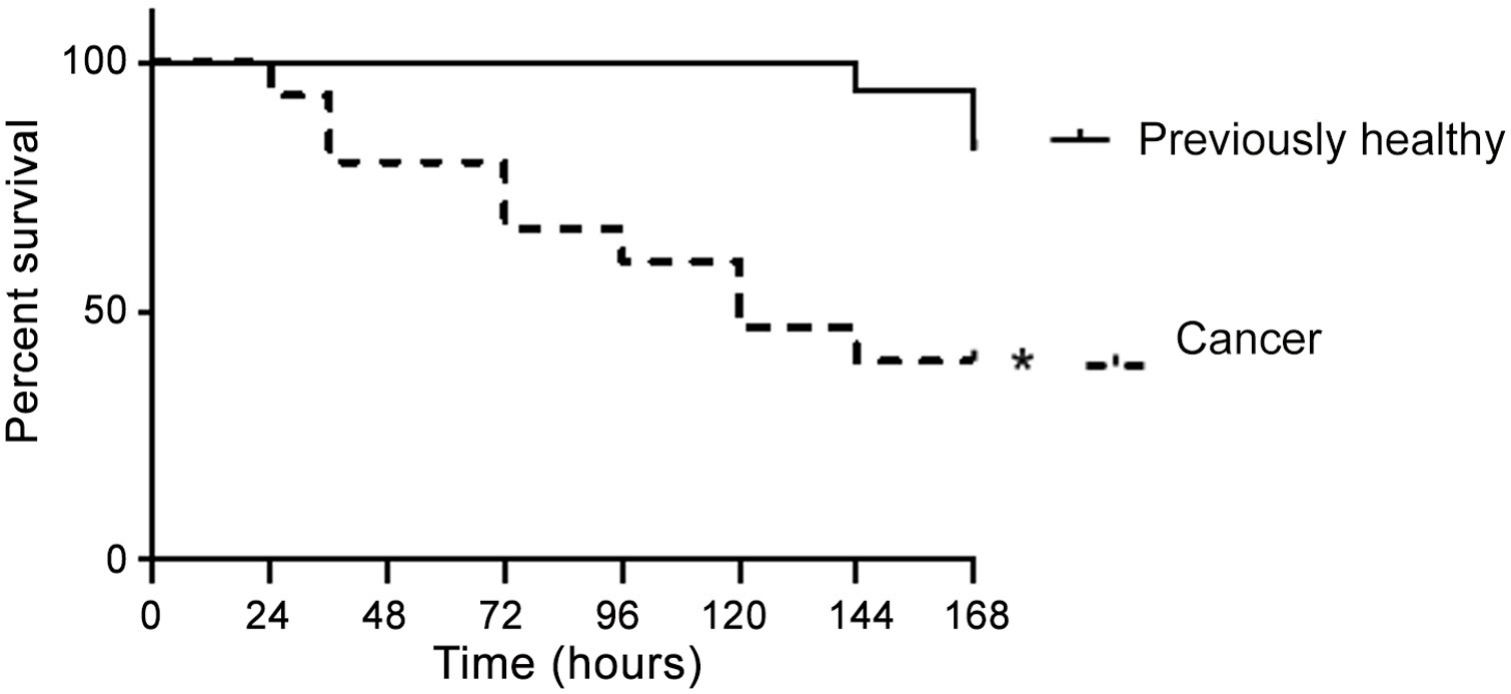
Effect of cancer on survival from sepsis. Previously healthy mice and those given an injection of LLC1 cells three weeks earlier (n = 15-17/group) were subjected to CLP. Cancer septic mice had significantly higher mortality than previously healthy septic mice (p = 0.003).

Seven-day mortality was 18% in previously healthy septic mice. In contrast, seven-day mortality was 60% in cancer septic mice (Fig 1).

### The presence of pre-existing lung cancer decreases CD4+ lymphocytes but not CD8+ lymphocytes following sepsis

**Fig 2:**
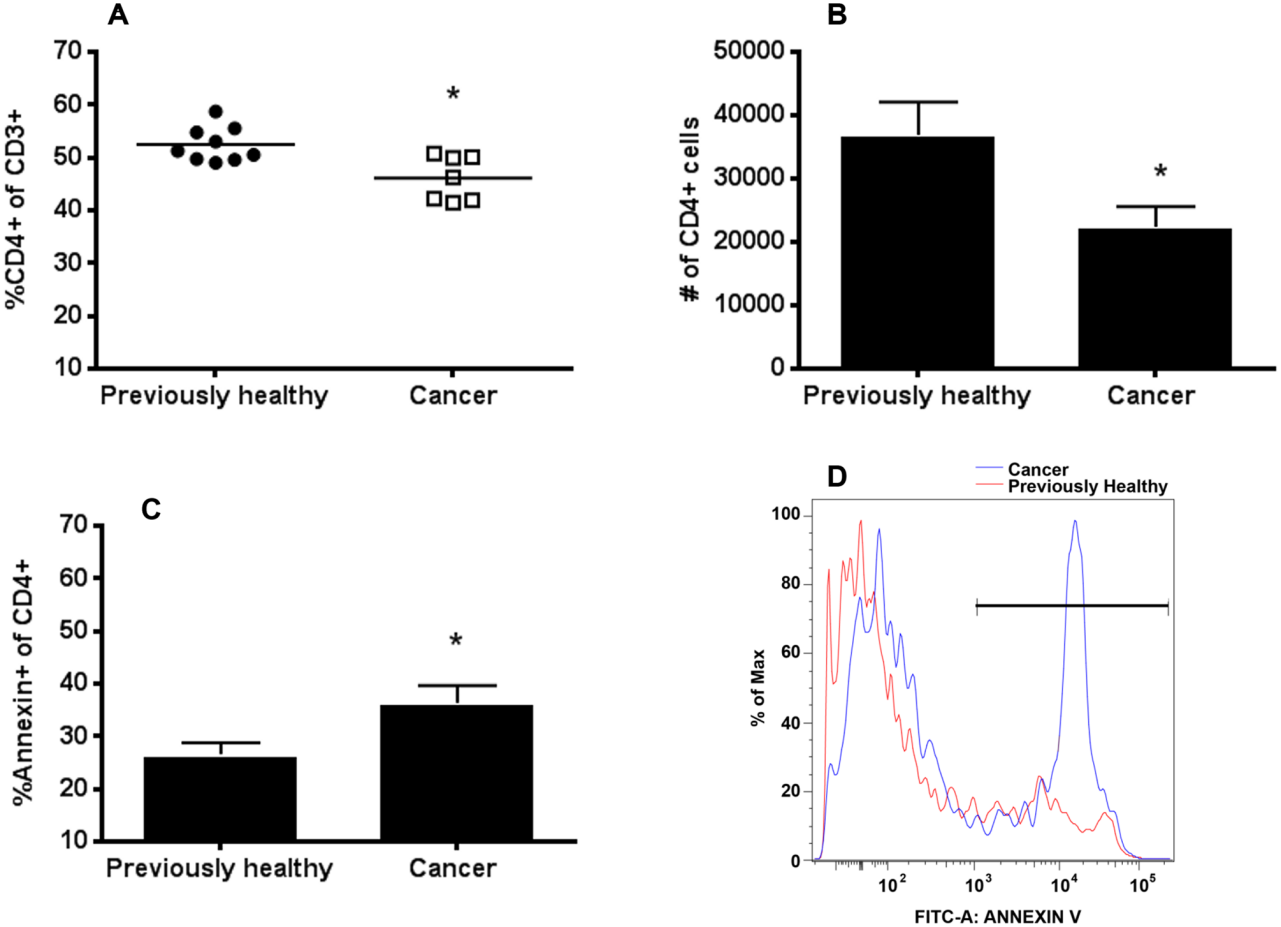
Effect of cancer on splenic CD4+ T cells following sepsis. Cancer septic mice had a significantly lower frequency of CD4+ T cells as a percentage of total CD3+ T cells (A, p = 0.02, n = 7–9) as well as a decrease in the total number of CD4+ T cells (B, p = 0.03, n = 8–10) compared to previously healthy septic mice. This was associated with an increase in annexin-positive CD4+ T cells in cancer septic mice (C, p = 0.04, n = 7–8). A representative flow cytometry histogram demonstrates increased annexin staining in cancer septic (blue) CD4+ T cells compared to previously healthy septic (red) CD4+ T cells (D).

**Fig 3:**
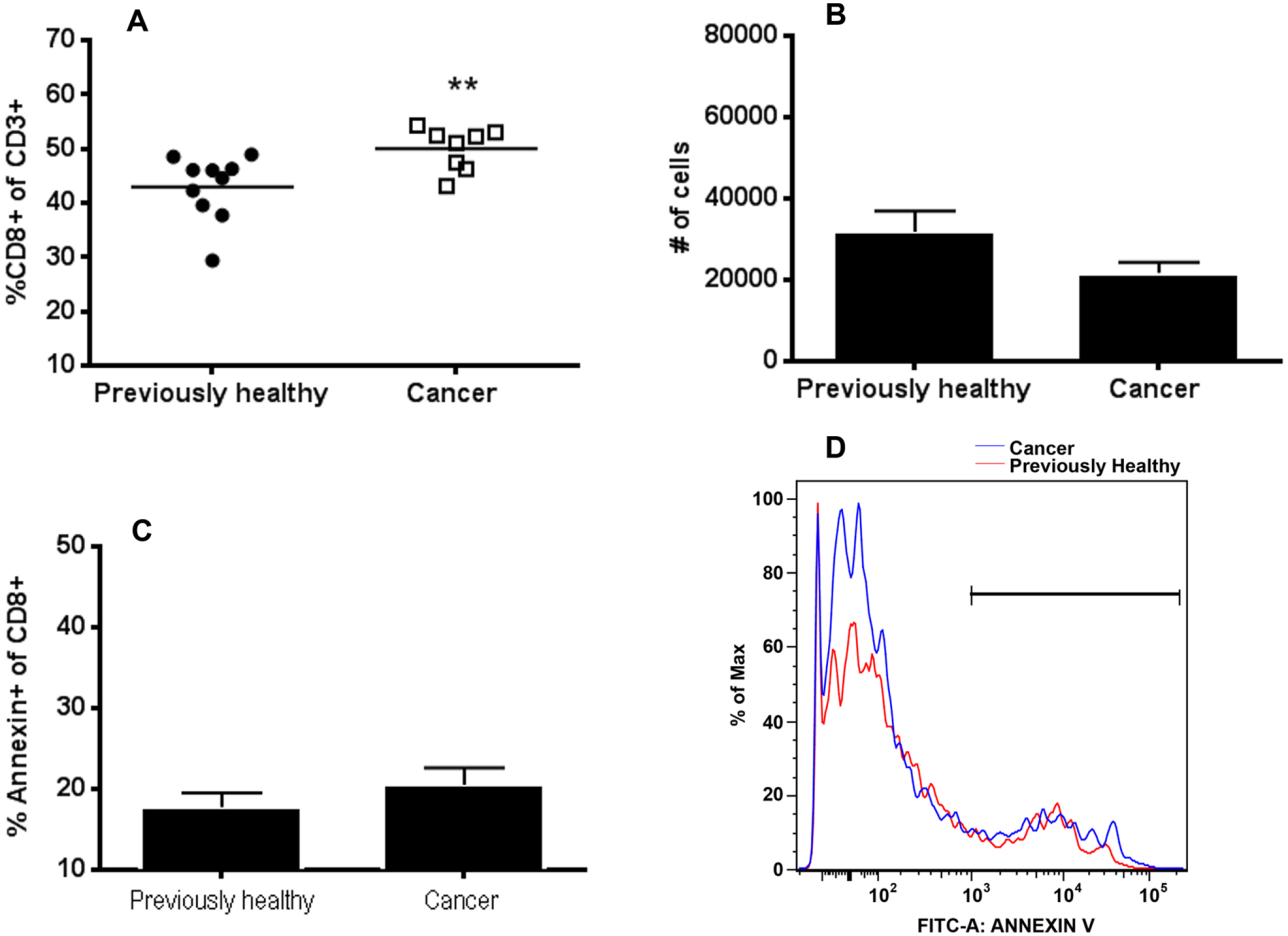
Effect of cancer on splenic CD8+ T cells following sepsis. Cancer septic mice had a significantly increased frequency of CD8+ T cells as a percentage of total CD3+ T cells (A, p = 0.007, n = 8–10). However, there was no statistically significant change in total number of CD8+ cells (B, p = 0.10, n = 8–10) or annexin-positive CD8+ T cells (C, p = 0.31, n = 7–10) suggesting the change in percentage of CD8+ cells was related to the decrease in CD4+ cells. A representative flow cytometry histogram demonstrates no difference in annexin staining in cancer septic (blue) and previously healthy septic (red) CD8+ T cells (D).

**Fig 4:**
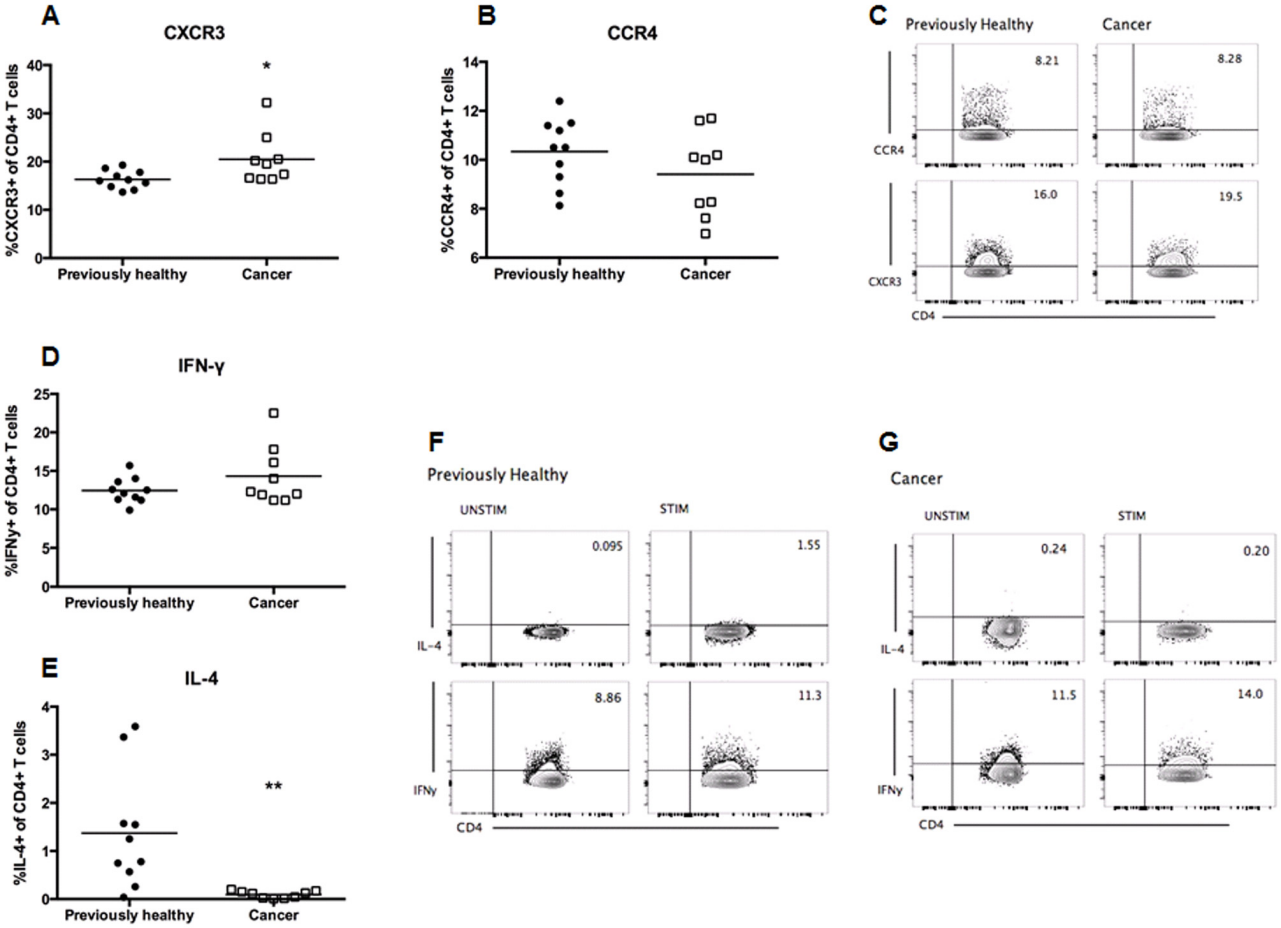
Effect of cancer on Th1 and Th2 markers and cytokine production following sepsis. Cancer septic mice had a modest increase in CD4+ T cell expression of the Th1 marker CXCR3 (A, p = 0.01, n = 9–10) but did not have a statistically significant change in expression of the Th2 marker CCR4 (B, p = 0.21, n = 9–10). A representative flow cytometry plot for both markers is included (C). Stimulated production of IFN-γ was not statistically different in CD4+ T cells from cancer septic mice (D, p = 0.17, n = 9–10); however, stimulated production of IL-4 was lower in CD4+ T cells from cancer septic mice (E, p = 0.006, n = 9–10). Representative flow cytometry plots for both unstimulated and stimulated IFN-γ and IL-4 are shown for previously healthy septic mice (F) and cancer septic mice (G).

Cancer septic mice had a decrease in both the frequency and absolute numbers of splenic CD4+ lymphocytes compared to previously healthy septic mice (Fig 2A and 2B). In addition, cancer septic mice had a higher frequency of annexin-positive staining CD4+ lymphocytes compared to previously healthy septic mice, suggesting increased apoptosis was responsible for the loss of CD4+ cells (Fig 2C and 2D). In contrast, while the frequency of splenic CD8+ lymphocytes was higher in cancer septic mice (Fig 3A), this was likely due to the decrease in CD4+ cells since no difference was seen in CD8+ cell numbers (Fig 3B) or annexin staining (Fig 3C and 3D) between cancer septic mice and previously healthy septic mice. CD4+ cell expression of the Th1 marker CXCR3 was increased in cancer septic mice (Fig 4A and 4C), while expression of the Th2 marker CCR4 was not different between previously healthy septic mice and cancer septic mice (Fig 4B and 4C). Stimulated production of Th1 effector cytokine IFN-γ by CD4+ T cells was not impacted by cancer (Fig 4D, 4F and 4G) while production of the Th2 effector IL-4 was significantly decreased in cancer septic mice (Fig 4E, 4F and 4G).

### The presence of pre-existing lung cancer decreases ability to clear local infection

**Fig 5:**
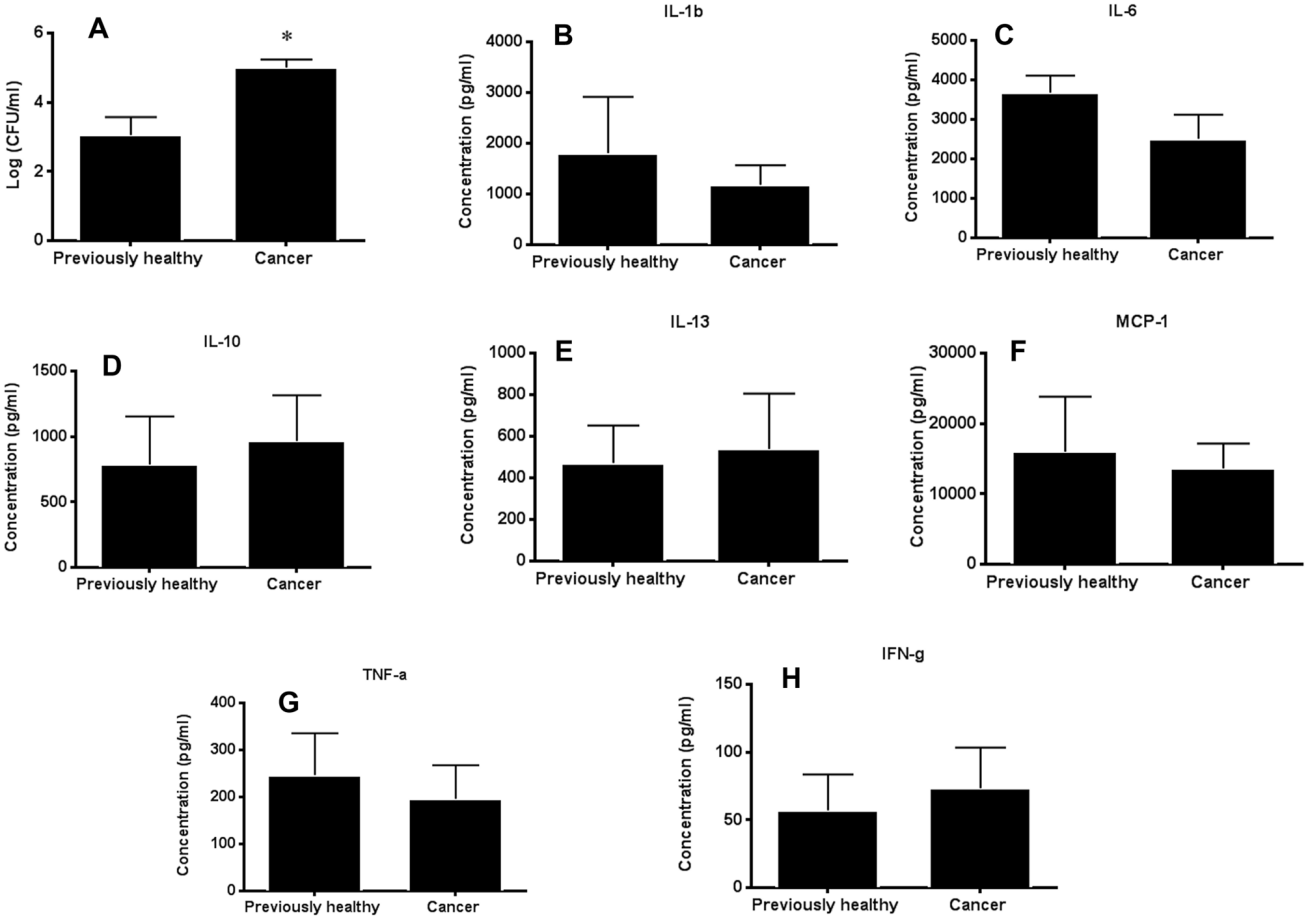
Effect of cancer on peritoneal bacteria and local inflammatory response following sepsis. Cancer septic mice had higher levels of bacteria in their peritoneal cavities than previously healthy septic mice (A, p = 0.005, n = 8–9). This change was not associated with differences in concentration of peritoneal IL-1β (p = 0.32), IL-6 (p = 0.18), IL-10 (p = 0.52), IL-13 (p = 0.97), MCP-1 (p = 0.67), TNF-α (p = 0.40), or IFN-γ (p = 0.27) (B-H, n = 8 for all groups).

**Fig 6:**
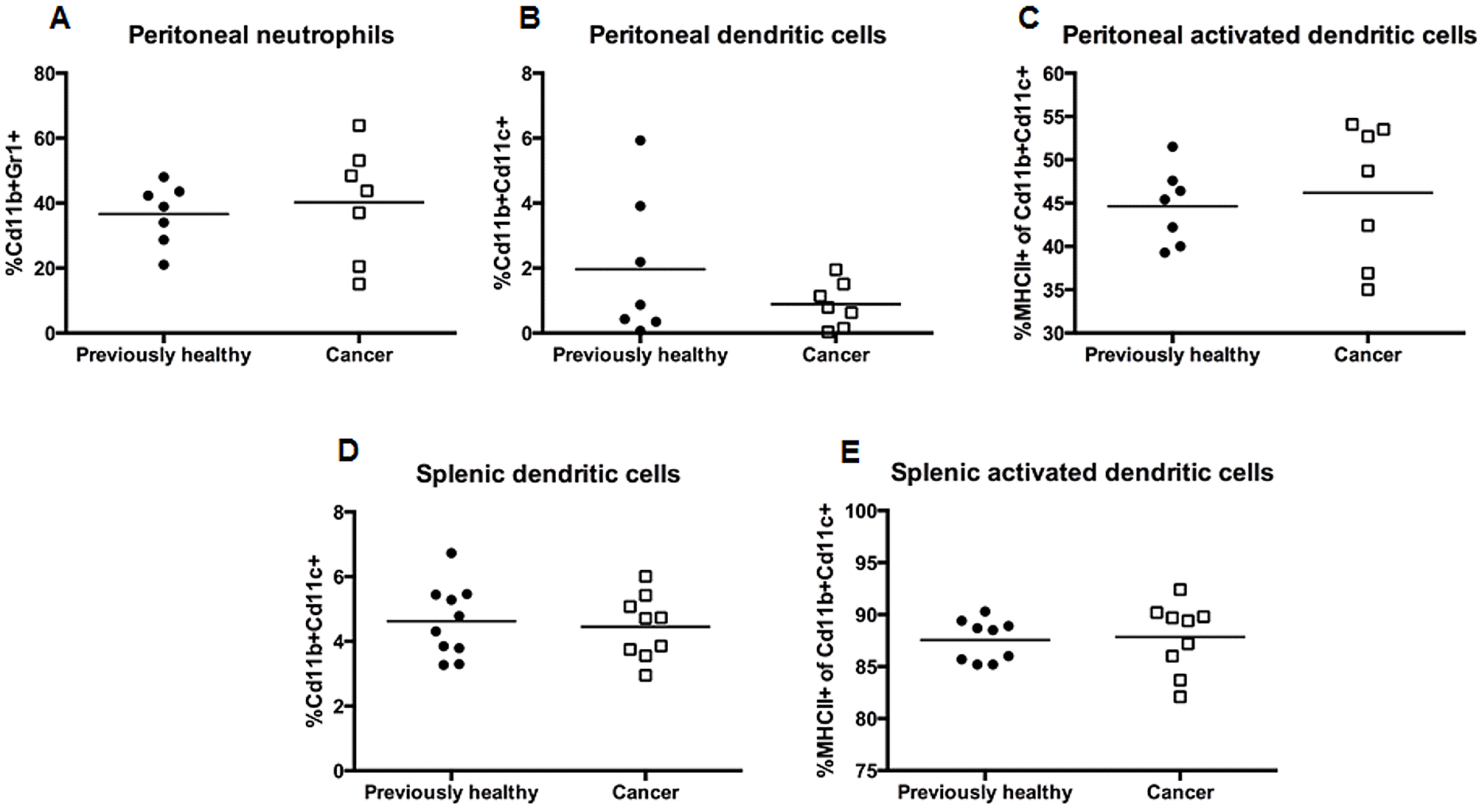
Effect of cancer on local neutrophil and dendritic cell responses following sepsis. Cancer septic mice and previously healthy septic mice had similar frequencies of neutrophils (A, p = 0.52, n = 7/group) and dendritic cells (B, p = 0.52, n = 7/group) in their peritoneal fluid, and there was no difference in the frequency of dendritic cell MHC II expression between the two groups (C, p = 0.52, n = 7/group). Cancer septic mice and previously healthy septic mice also had similar frequencies of dendritic cells (D, p = 0.73, n = 9–10) and activated dendritic cells (E, p = 0.83, n = 9–10) in splenocytes.

Peritoneal bacterial burden was higher in cancer septic mice than previously healthy septic mice (Fig 5A). This was not associated with changes in Interleukin (IL)-1β, IL-6, IL-10, IL-13, MCP-1, TNF-α, or IFN-γ in the peritoneal fluid (Fig 5B–5H). Peritoneal fluid also contained similar percentages of neutrophils (Fig 6A) and dendritic cells (Fig 6B) between previously healthy septic mice and cancer septic mice. Dendritic cell activation as determined by MHC II expression on cells from peritoneal fluid was also similar between the groups (Fig 6C). Splenic dendritic cell frequency and activation were also unaffected by the presence of cancer (Fig 6D and 6E). There were no differences in absolute numbers of neutrophils or dendritic cells (data not shown).

**Fig 7:**
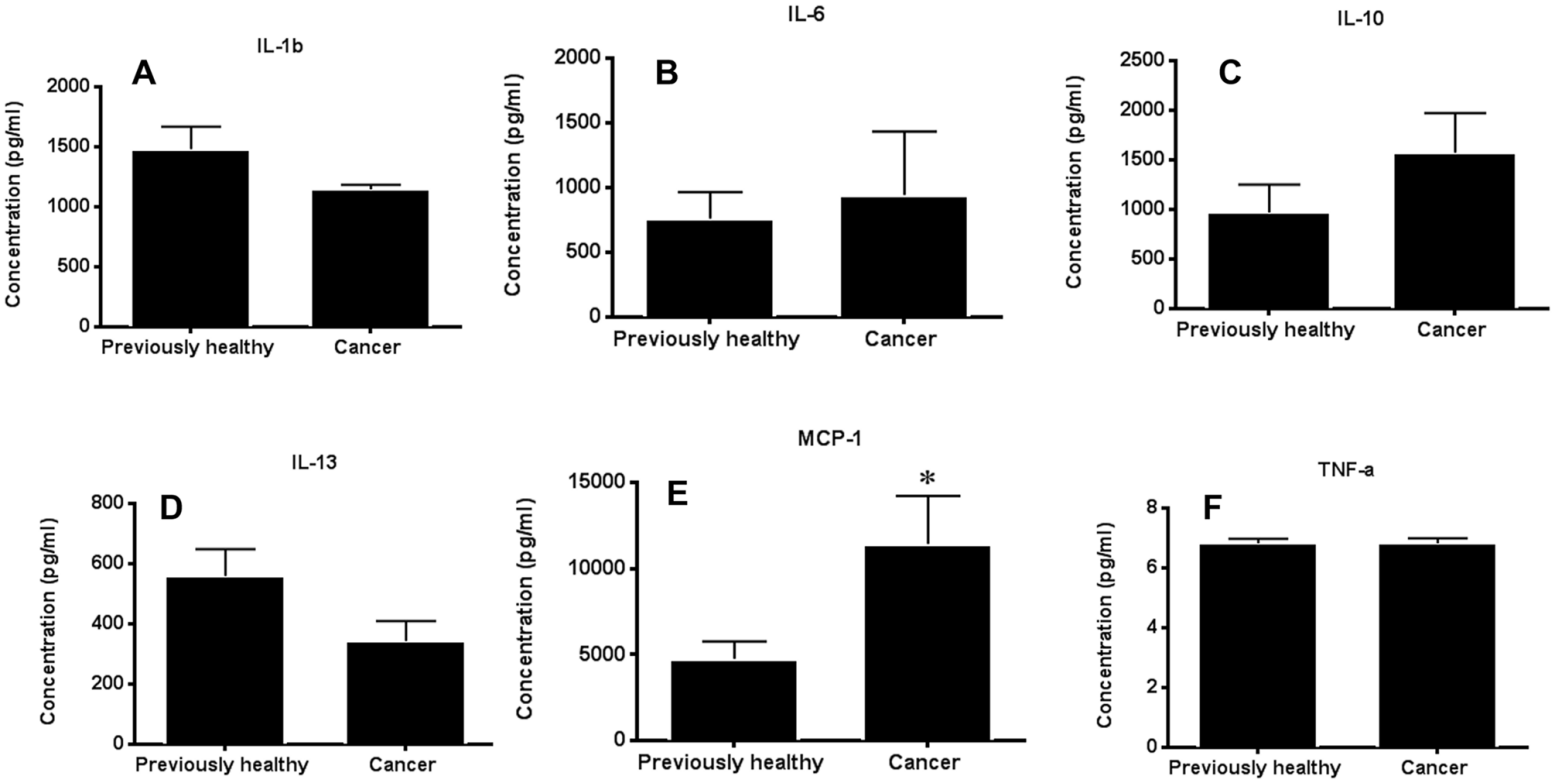
Effect of cancer on systemic cytokines following sepsis. No significant differences were noted in IL-1β (p = 0.10), IL-6 (p = 0.63), IL-10 (p = 0.24), IL-13 (p = 0.07), or TNF-α (p = 0.99) (B-F, n = 7-8/group). In contrast, increased MCP-1 was detected in the serum of cancer septic mice compared to previously healthy septic mice (E, p = 0.04, n = 7–8).

Serum cytokines were similar between cancer septic mice and previously healthy septic mice except for an increase in MCP-1 in cancer septic mice (Fig 7). No difference in bacterial burden was identified in quantitative blood cultures between previously healthy septic mice and cancer septic mice (data not shown).

### The presence of pre-existing lung cancer decreases crypt proliferation but does not alter other components of intestinal integrity following sepsis

**Fig 8:**
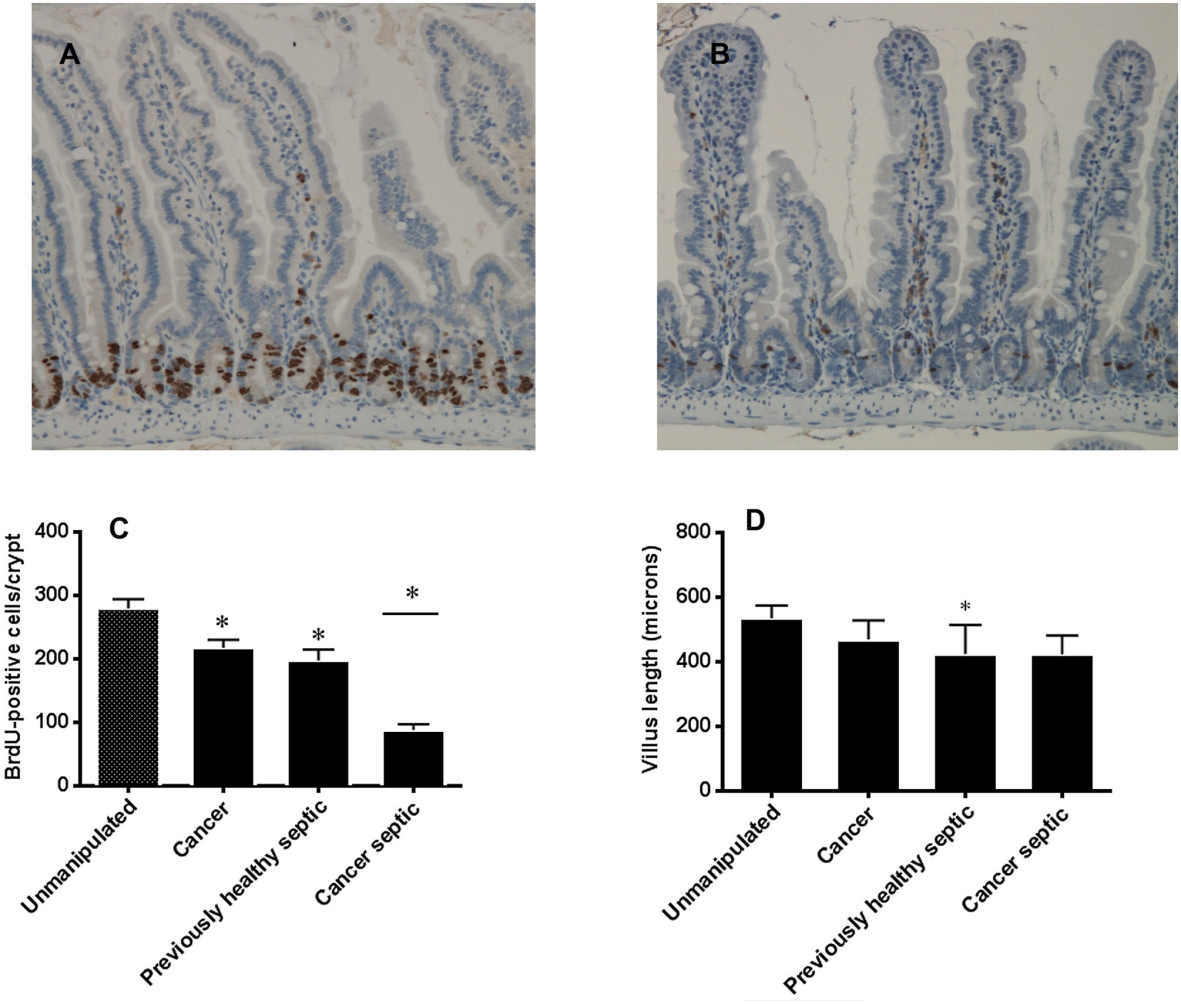
Effect of cancer on intestinal proliferation following sepsis. Previously healthy septic mice (A) had qualitatively higher levels of proliferation than cancer septic mice (B, BrdU-positive crypt cells stain brown). Quantitatively, both cancer in isolation and sepsis in isolation decrease crypt proliferation (C, p = 0.02 and 0.008 respectively compared to unmanipulated mice). The combination of cancer and sepsis further decreased intestinal proliferation, moreso than was seen with either variable in isolation (p<0.001 previously healthy septic vs. cancer septic, n = 8–10 for all groups in panel C). In contrast, while villus length was decreased by sepsis (but not cancer) in isolation (p = 0.008), there was no difference in villus length between previously healthy septic and cancer septic mice (D, p>0.99, n = 8–9 for all groups).

Cancer in isolation decreases crypt proliferation compared to unmanipulated mice. Sepsis in isolation also decreases crypt proliferation compared to unmanipulated mice. The combination of cancer and sepsis causes a further disproportionate decrease in crypt proliferation compared to sepsis alone as cancer septic mice have lower proliferation than previously healthy septic mice (Fig 8A–8C).

In contrast, villus length is not affected by cancer in isolation, is decreased as has been previously shown by sepsis (26), but is not different between previously healthy septic mice and cancer septic mice (Fig 8D). Cancer and sepsis also did not impact intestinal permeability or crypt apoptosis compared to sepsis alone (data not shown).

### The presence of pre-existing lung cancer worsens biochemical kidney function without causing liver injury following sepsis

**Fig 9:**
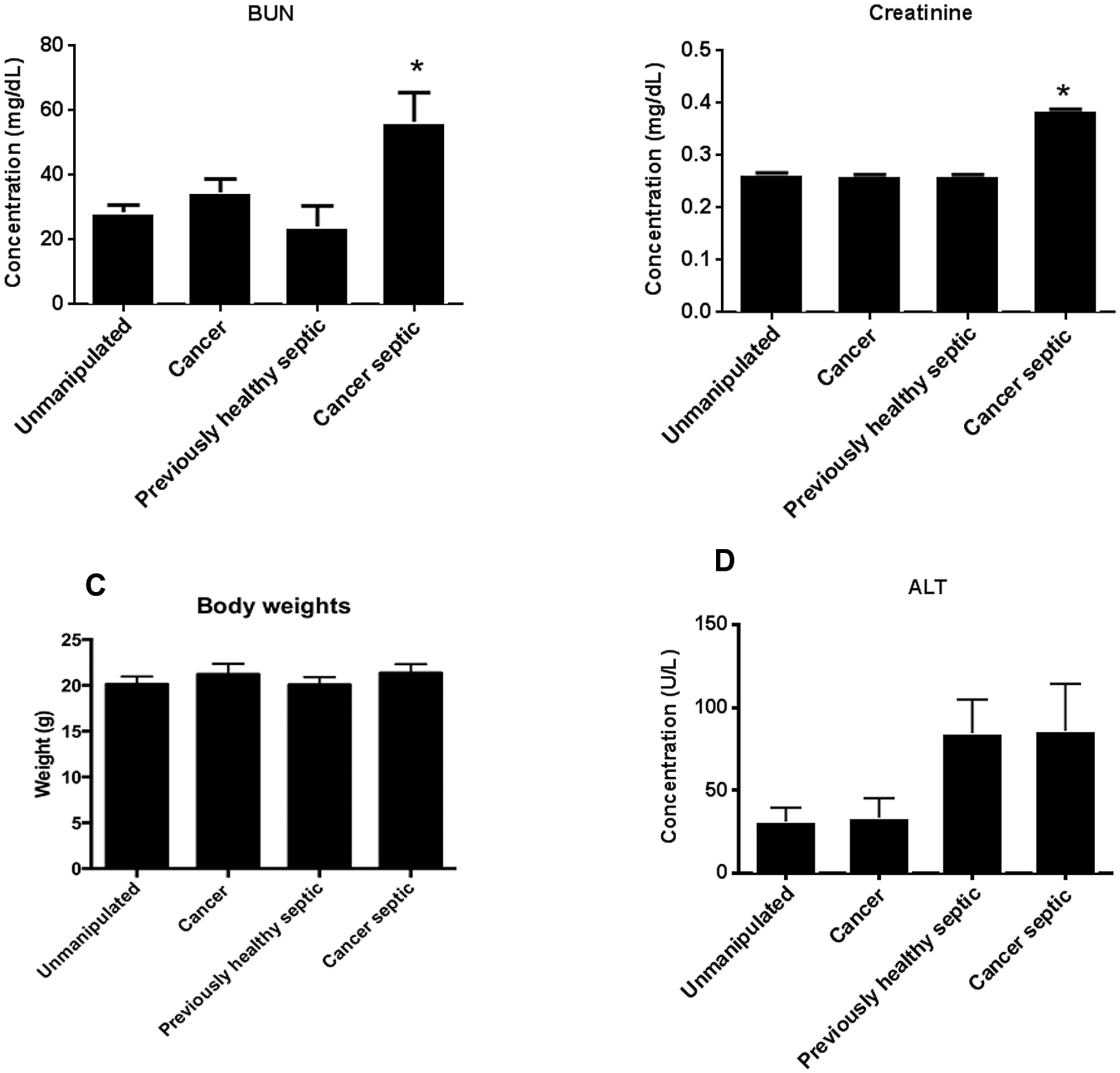
Effect of cancer on renal and liver function following sepsis. Neither cancer nor sepsis in isolation impacted BUN or Cr levels (A, B). However, the combination of both insults worsens renal function as both BUN and Cr were higher in cancer septic mice than previously healthy septic mice (p = 0.004 and p<0.0001 respectively, n = 8 for all groups). Body weights were similar in all groups, regardless of the presence of cancer or a septic insult 24 hours earlier (C). While cancer had no impact on liver function as assayed by ALT, sepsis, in isolation, increased ALT compared to unmanipulated mice (D, p<0.001). However, this was not worsened by malignancy as cancer septic mice and previously healthy septic mice had similar ALT levels (p>0.99, n = 8–10 for all groups).

Neither cancer nor sepsis in isolation affected renal function as BUN and Cr levels were similar in unmanipulated, cancer and previously healthy septic mice. In contrast, both BUN and Cr were higher in cancer septic mice (Fig 9A and 9B); however kidneys appeared grossly normally histologically (data not shown). No differences in body weight were identified between previously healthy septic mice and cancer septic mice (Fig 9C).

Basal levels of ALT were similar in unmanipulated and cancer mice. While sepsis increased serum ALT levels, this was not affected by the presence of cancer since ALT was similar between cancer septic and previously healthy septic mice (Fig 9D). Further, there were no differences in liver histology between any of the groups (data not shown).

### The presence of pre-existing lung cancer decreases BAL MPO and increases BAL protein following sepsis

**Fig 10:**
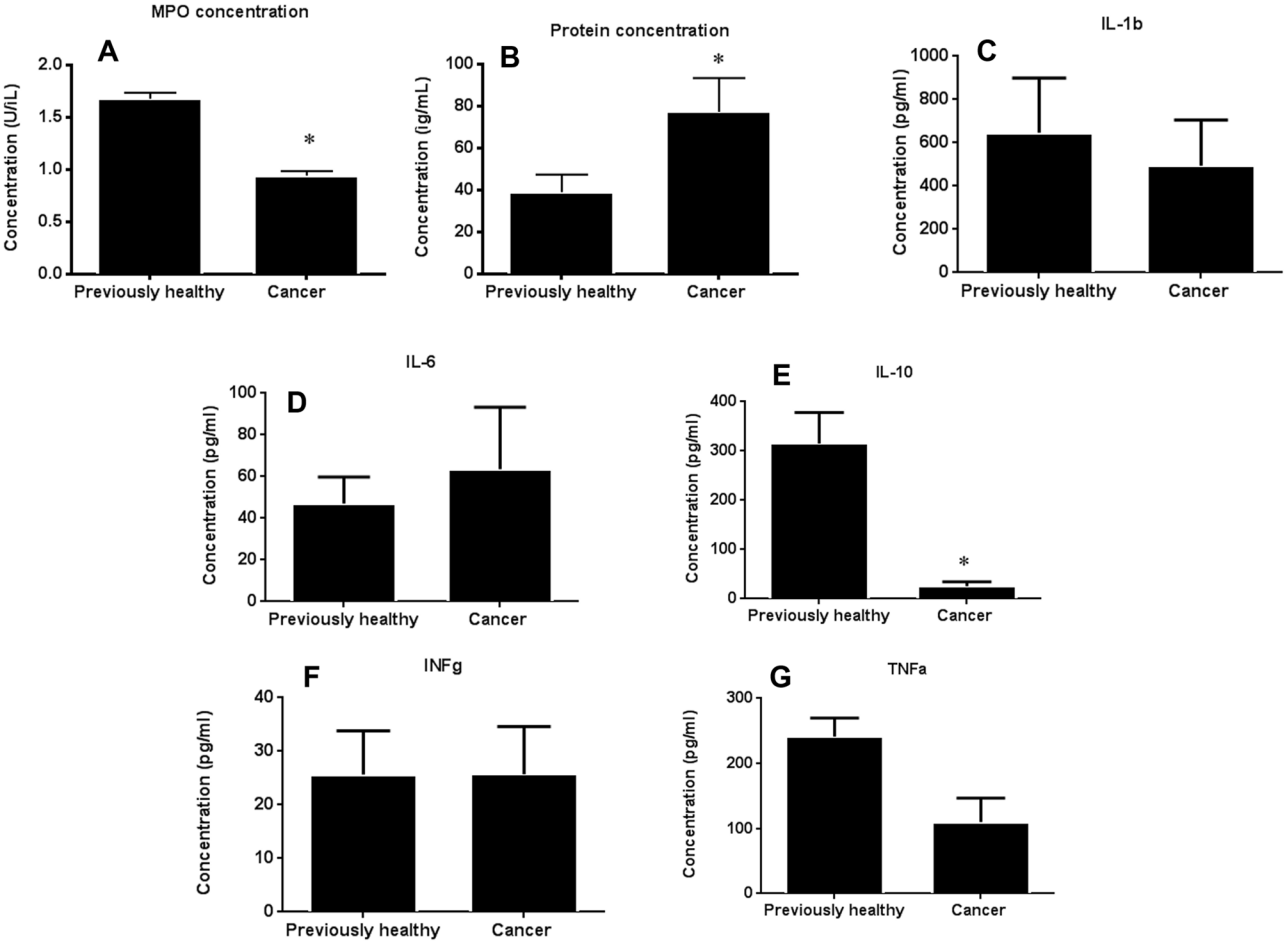
Effect of cancer on lung inflammation following sepsis. MPO activity in BAL fluid was decreased in cancer septic mice compared to previously healthy septic mice (A, p<0.0001, n = 8). In contrast, protein concentration in BAL fluid was higher in cancer septic mice (B, p = 0.04, n = 6–8). BAL cytokines were similar between mice for IL-1β (p = 0.47), IL-6 (p = 0.59), IFN-γ (p = 0.99) and TNF-α (p = 0.11) but IL-10 levels were lower in cancer septic mice (p = 0.0005, n = 9–10 for all groups).

MPO activity was lower in BAL fluid in cancer septic mice compared to previously healthy septic mice (Fig 10A). In contrast, BAL protein was higher in cancer septic mice compared to previously healthy septic mice (Fig 10B). BAL cytokines were generally independent of the presence of cancer although BAL IL-10 was lower in cancer septic mice (Fig 10C–10G). Despite differences noted in BAL fluid, no differences were identified in inflammation score or percentage of inflammatory cells between cancer septic and previously healthy septic mice on histologic examination (data not shown).

### The presence of pre-existing lung cancer does not alter complete blood counts following sepsis

**Fig 11:**
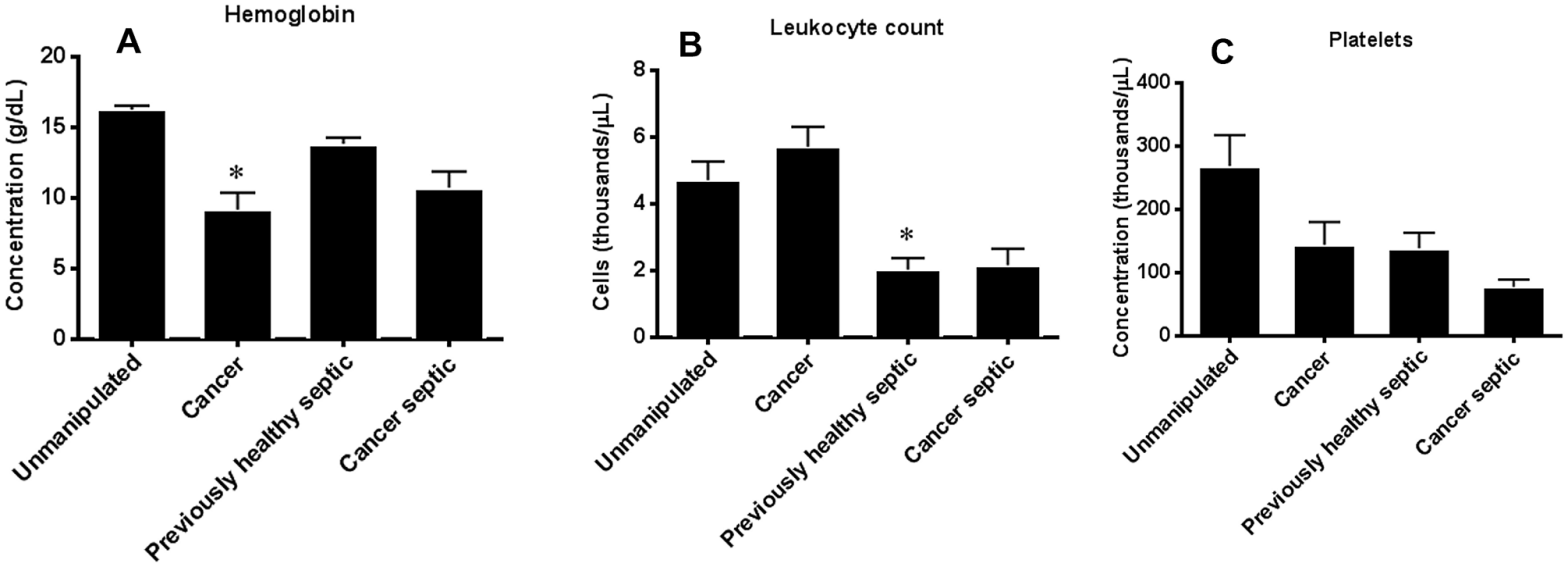
Effect of cancer on hemoglobin, leukocyte count and platelet count following sepsis. Cancer, in isolation, decreased serum hemoglobin levels (A, p<0.0001); however, this was not impacted by sepsis and there was no statistically significant difference between cancer septic and previously healthy septic mice (p = 0.14, n = 5-9/group). In contrast, while cancer did not impact leukocyte count (B), sepsis, in isolation caused a decrease in in leukocyte count (p = 0.02). No statistically significant difference in leukocyte count was seen between cancer septic mice and previously healthy mice (p = 0.99, n = 5-9/group). Platelet count was similar between unmanipulated mice and those with cancer or sepsis in isolation (C). No statistically significant difference in platelet count was seen between cancer septic mice and previously healthy mice (p = 0.78, n = 5-9/group).

Cancer, in isolation, causes anemia as hemoglobin levels were significantly lower in cancer mice than unmanipulated mice (Fig 11A). However, sepsis, in isolation did not alter hemoglobin levels and there was no statistically significant difference in hemoglobin levels between previously healthy septic mice and cancer septic mice. Cancer, in isolation, did not change total leukocyte count although sepsis, in isolation, decreased total leukocyte count (Fig 11B). The combination of sepsis and cancer did not alter total leukocyte count further since it was similar between previously healthy septic mice and cancer septic mice. Neither cancer nor sepsis in isolation led to a statistically significant change in platelet count compared to unmanipulated animals, and similarly, there were no statistically significant differences in platelet count between previously healthy septic mice and cancer septic mice (Fig 11C).

## Discussion

Similar to the human condition and our previous mouse model of cancer followed by sepsis, this study demonstrated that the presence of a malignancy significantly worsens survival from sepsis. However, parameters associated with increased mortality varied greatly between mice injected with lung cancer cells followed by CLP in this study and our prior findings of mice injected with pancreatic cancer cells followed by *Pseudomonas aeruginosa* pneumonia. In this study, we noted increased apoptosis in splenic CD4+ T lymphocytes, decreased crypt proliferation, decreased local infection clearance, increased systemic MCP-1, worse renal function, lower BAL MPO activity, higher BAL protein and lower BAL IL-10 in cancer septic mice compared to previously healthy septic mice. In contrast, in mice with pancreatic cancer cells followed by *Pseudomonas aeruginosa* pneumonia, we found decreased apoptosis in both T and B lymphocytes, increased gut epithelial apoptosis, increased bacteremia without alterations in local infection, and higher BAL IL-6 and IL-10 compared to previously healthy septic mice. Remarkably, except for elevated mortality, there was essentially no overlap in the associated abnormalities between lung cancer/CLP and pancreatic cancer/pneumonia mice.

Whether a common host response exists in sepsis is controversial [27–29]. Our results can be interpreted that the mechanisms responsible for mortality vary significantly, at least in part, with tumor type and sepsis model. Alternatively, since our results are associative, it is possible that none of the abnormalities detected in either model of cancer and sepsis is actually responsible for the increased mortality. While we cannot rule out that possibility, it is reasonable to attempt to put the findings described herein into context of existing literature.

The concept that T lymphocyte derangements play a crucial role in mediating mortality from sepsis is now over a decade old. Multiple studies demonstrate that preventing T cell apoptosis improves survival following CLP [30–32]. The findings that a) CD4+ lymphocytes are decreased and b) Annexin V staining is increased are consistent with a more profound immunosuppressive state in mice with lung cancer followed by CLP compared to previously healthy mice subjected to the same insult. While this would typically be thought of as maladaptive, it should be noted that prevention of lymphocyte apoptosis worsened survival in mice overexpressing Bcl-2 in lymphocytes as well as in BIM knockouts following pneumonia in mice with pancreatic cancer [15], so the functional significance of increased CD4+ lymphocyte apoptosis in cancer septic mice needs to be examined further in future experiments.

Altered Th1 and Th2 responses have also been described in sepsis [17,33], and it seemed plausible that cancer-mediated increases in T cell exhaustion could potentially induce a shift towards a Th2 response during sepsis [12]. However, our results do not neatly fit this hypothesis. Although we demonstrated a modest increase in expression of CXCR3 on CD4+ T cells in cancer septic mice (which may suggest a skewing towards a Th1 response), the biological significance of this is unclear since we found no associated differences in production of the Th1 effector IFN-γ by CD4+ T cells. In addition, we did not detect a difference in expression of the Th2 marker CCR4 but did observe decreased production of the Th2 effector IL-4 from CD4+ cells taken from cancer septic mice. These somewhat conflicting data suggest that while there may be imbalances in the Th1/Th2 response following sepsis in animals with cancer, the changes are likely not a simple shift directly toward one phenotype or another, and the degree to which those changes impact mortality is yet to be determined.

There were significantly higher levels of bacteria present in the peritoneal cavity 24 hours after CLP in cancer septic mice. Even though local cytokine, neutrophil, and dendritic cell profiles were similar between cancer septic and previously healthy septic mice, it is plausible that the basal immunosuppressive state caused by sepsis predisposes animals to increased local infection. How this suppression is functionally enacted requires further investigation in future studies. Notably, polymicrobial sepsis, as would typically be seen following fecal peritonitis, is associated with higher mortality in patients with cancer and sepsis[9].

Gut integrity is altered by sepsis, with increased apoptosis and permeability as well as decreased proliferation and villus length. These and other alterations in gut integrity can result in distant organ injury and propagation of systemic inflammation, resulting in the hypothesis that the gut is the "motor" driving critical illness [16,34,35]. Although multiple parameters of gut integrity were altered by sepsis in isolation in this study, the majority of these were similar between cancer septic mice and previously healthy septic mice. One exception was gut proliferation, which was decreased by both cancer and sepsis in isolation but disproportionately decreased by the combination of both insults. In light of the complex function and architecture of the gut, it is possible that the marked decrease in gut proliferation seen in cancer septic mice resulted in downstream functional changes within the intestine, resulting in either local or distant injury. Since the gut is a continuously renewing organ that replaces itself on average every 3–5 days[35], future experiments are required to determine how long the decreased proliferation induced by the combination of sepsis and cancer persists.

Both BUN and Cr (markers of renal dysfunction) are similar in unmanipulated mice, cancer mice and previously healthy septic mice, suggesting neither cancer nor sepsis impacts renal function. However, BUN and Cr are statistically higher in cancer septic mice compared to previously healthy septic mice. We do not believe that the modest degree of biochemical acute kidney injury seen at 24 hours is solely responsible for mortality seen in cancer septic mice, and it is questionable if the small absolute difference in serum BUN/creatinine values is biologically meaningful, especially given the absence of histologic abnormalities in the kidneys in all groups. It is possible that renal function worsens throughout the course of sepsis in this model and therefore contributes to mortality more significantly at later time points. Of note, the etiology of renal dysfunction that is seen exclusively in cancer septic mice remains to be determined.

Finally, MPO activity, protein and IL-10 were all altered in BAL fluid of cancer septic mice although this was not accompanied by differences in histologic lung inflammation or other BAL cytokines between previously healthy septic mice and cancer septic mice. While lowered MPO activity and decreased levels IL-10 may suggest a dampened pulmonary inflammatory response, more detailed assays would be needed to determine if this is actually the case. In addition, since studies have shown that pulmonary disease does not represent a significant cause of death in mice subjected to CLP[36], the functional significance of these findings is unclear.

This study has a number of limitations. While a number of abnormalities were identified that are associated with increased mortality, we cannot conclude that any of them are causative without performing additional mechanistic studies. Next, all non-survival studies were performed at a single timepoint (24 hours), so it is likely that our experimental design missed temporal trends that would be important towards understanding the relationship between cancer and sepsis, especially since there appear to be different inflammatory states depending on how far out a host is from their septic insult [37]. Next, although we have attempted to compare our results to our previous study on pancreatic cancer/pneumonia, there are three variables that are different in this manuscript—type of cancer, sepsis model and the fact that micro-metastatic disease was noted in this study with LLC-1 cells whereas Pan02 pancreatic cancer cells are not associated with metastatic disease. To determine which of these are most responsible for differences, changing only a single variable at a time would be required. Next, tumor cells were injected into the thigh of murine recipients and thus do not replicate the development of an *in-situ* lung cancer. Mice with naturally occurring tumors could potentially be exposed to local tumor-related factors that cause a different inflammatory milieu than is seen in our model. Further a more gradual tumor onset growth could potentially alter dynamics of tumor-immune cell interaction, leading to chronic inflammatory changes not seen after only three weeks of tumor growth. Finally, tumors tend to develop in aged patients, whereas this study examined mice that were 9–12 weeks old prior to the onset of sepsis. It is unclear how well young mice function as surrogates for aged patients, and our experimental design precluded us from assessing the impact of age on the pathophysiology of cancer and sepsis.

Despite these limitations, our data yield new insights into a clinically relevant model of both a common cancer and a common cause of sepsis, and the interplay between the two insults. Further research is required to determine if the multiple pathophysiologic abnormalities identified herein are important in mediating the increased mortality seen when sepsis occurs in the setting of cancer, and the role of specific tumors or types of sepsis in mediating this complex interaction.
